# Editorial: Breakfast around the globe: habits, effects, and novel food for thought

**DOI:** 10.3389/fnut.2024.1485384

**Published:** 2024-09-18

**Authors:** Tobias Otterbring, Stacey Finkelstein, Michal Folwarczny

**Affiliations:** ^1^University of Agder, Kristiansand, Norway; ^2^Stony Brook University, Stony Brook, NY, United States; ^3^Discipline of Marketing, J.E. Cairnes School of Business and Economics, University of Galway, Galway, Ireland

**Keywords:** breakfast, hunger, breakfast skipping, dietary recall, healthy eating concerns, executive functions, cardiometabolic health

Breakfast habits have mirrored economic, cultural, and societal changes from ancient Egypt, where breakfast was both a necessity and a ritual, to modern-day cosmopolitan societies embracing fusion cuisines and personalized nutrition trends (1). The importance placed on breakfast varies enormously across cultures. For instance, whereas many people living in Western cultures often share the belief that breakfast is the most important meal of the day, Japanese consumers typically consume twice as many calories at dinner as at breakfast, suggesting that the last meal is at least equally important for these individuals (2).

There are also many misconceptions about the health impact of breakfast composition. For example, despite consuming half of the daily recommended dose of sugar at breakfast, four out of five parents in the UK believe their children's breakfast is healthy (3). Meanwhile, breakfast composition is evolving globally, with traditional items losing popularity to convenience foods like breakfast bars or high-protein yogurts (4). This shift coincides with a growing interest in habitual and planned breakfast skipping. A YouGov (5) survey found that a quarter of Americans have tried the 16:8 intermittent fasting diet, typically involving breakfast skipping, with half of these individuals finding it very effective for weight loss. However, breakfast skipping might also have certain costly consequences, with people who abstain from their morning meals becoming both hungrier (6, 7) and “hangrier” (8, 9), while simultaneously prioritizing present pleasures and disregarding future-focused benefits (10, 11). Accordingly, despite the potential for intermittent fasting diets to reduce body mass, more research is needed to understand the effects of these emerging eating patterns on factors such as cognitive functioning, food choices later in the day, and psychological wellbeing, among others.

The current Research Topic delves into the multifaceted role of breakfast in contemporary society, examining its nutritional, cultural, and psychological influences. By bringing together diverse perspectives and recent research, this Research Topic aims to provide valuable insights for academics, nutritionists, and the general public, highlighting the evolving nature of breakfast, its composition, and its significance in our daily lives. The included articles cover topics such as health risks related to breakfast frequency, the links between breakfast patterns and cognitive functioning, the influence of hunger on food choices, and the nutritional quality of breakfasts. The studies involve data from diverse samples, including Peruvian university teachers, adolescents in Shanghai, children in France, and consumers in Scandinavia, highlighting the cross-cultural scope of this Research Topic.

Poinsot et al. leverage dietary recall to better understand French children's consumption of food at breakfast. Using this approach, they triangulate on breakfast quality, exploring relative choices of dairy, carbohydrates, protein, etc. They describe how household income and food insecurity impact breakfast quality, thus highlighting an important structural barrier to consuming healthier meals.

Otterbring et al. find that healthy eating concerns moderate the impact of hunger (e.g., due to breakfast skipping) on the likelihood of choosing indulgent foods (foods that are relatively higher in sugar, calories, or fat) among consumers in Scandinavia. Their results suggest that the impact of hunger on indulgent food choices may only hold under certain circumstances.

Wang et al. explore the link between breakfast patterns and executive function among a large sample of Chinese adolescents. They find that adolescents who skip breakfast tend to have greater executive function difficulties than their counterparts who eat breakfast, with adolescents who have an abundance of food for breakfast being at a particularly low risk of having executive function difficulties. These findings underscore the importance of breakfast for cognitive functioning.

Saintila et al. investigate the link between the frequency of breakfast consumption and cardiometabolic risk in Peruvian university teachers. Their results suggest that breakfast skipping for several days per week might have harmful effects on cardiometabolic health, thus implying that promotional messages about the health gains of eating breakfast might benefit the public.
